# A Confirmation for the Positive Electric Charge of Bio-Molecular Motors through Utilizing a Novel Nano-Technology Approach In Vitro

**DOI:** 10.3390/ijms21144935

**Published:** 2020-07-13

**Authors:** Mitra Shojania Feizabadi, Ramiz S. Alejilat, Alexis B. Duffy, Jane C. Breslin, Ibukunoluwa I. Akintola

**Affiliations:** Department of Physics, Seton Hall University, South Orange, NJ 07079, USA; ramiz.alejilat@student.shu.edu (R.S.A.); alexis.duffy@student.shu.edu (A.B.D.); jane.breslin@student.shu.edu (J.C.B.); ibukunoluwa.akintola@student.shu.edu (I.I.A.)

**Keywords:** microtubule, biomolecular motors, kinesin-1, electro-conduction, nanotechnology

## Abstract

Molecular motors are microtubule-based proteins which contribute to many cell functions, such as intracellular transportation and cell division. The details of the nature of the mutual interactions between motors and microtubules still needs to be extensively explored. However, electrostatic interaction is known as one of the key factors making motor-microtubule association possible. The association rate of molecular motors to microtubules is a way to observe and evaluate the charge of the bio-motors in vivo. Growing evidence indicates that microtubules with distinct structural compositions in terms of beta tubulin isotypes carry different charges. Therefore, the electrostatic-driven association rate of motors–microtubules, which is a base for identifying the charge of motors, can be more likely influenced. Here, we present a novel method to experimentally confirm the charge of molecular motors in vitro. The offered nanotechnology-based approach can validate the charge of motors in the absence of any cellular components through the observation and analysis of the changes that biomolecular motors can cause on the dynamic of charged microspheres inside a uniform electric field produced by a microscope slide-based nanocapacitor. This new in vitro experimental method is significant as it minimizes the intracellular factors that may interfere the electric charge that molecular motors carry.

## 1. Introduction

Biomolecular motors are one of the cellular components carrying a significant role in cell functions [1,2]. Single molecule biophysical studies have made key contributions in understanding the intracellular functions of motors. Extensive studies in this field have revealed the diverse roles that molecular motors carry, ranging from cellular transportation to mitosis and cell migration. Kinesin motors are one group of molecular motors with a large number of members. These motors work in association with microtubules, using them as their tracks to carry out their cellular tasks [3].

Some members of the kinesin family, such as kinesin-1, are known for their ability to move along microtubules [4,5,6]. These motors mainly contribute to cellular transportation. In contrast, some members, such as kinesin-5, are poorly processive, but their functions are highlighted due to the role that they play in the process of cell division and chromosome segregation [7].

New evidence indicates that the functions of motors can be regulated by the structural specifications of microtubules that they employ as their track. The changes in biomechanical specifications of molecular motors, including processivity, velocity, and their binding time to microtubules, which has been reported in some studies, strengthen the possibility that the function of molecular motors can be cell specific [8,9,10,11,12]. The distinct interaction of motors with microtubules is suggested as one of the reasons that can potentially explain the different behavior that molecular motors express along structurally diverse microtubules [9,12,13]. However, the underlying mechanism of such motor–microtubule interactions are poorly understood. Some findings propose that, among several possible factors, electrostatic interaction between molecular motors and microtubules may have a critical role in regulating the motors’ functions [14]. The structural diversity of microtubules in terms of beta tubulin isotypes and the different amount of charge that their C-terminal tails carry is described as one of the sources of different electrostatic interactions [9,15,16]. Currently, most of the reported studies have mainly concentrated on the different electric charges of tubulin, while less attention has been given to achieve a better understanding of the electrostatic charge that molecular motors carry [17,18,19,20]. While there are some reported methods to determine the charge of proteins, our knowledge about identifying the charge of molecular motors is mainly based on the different association rates they express in conjunction with microtubules in vivo [13,21,22]. However, these rates can possibly be impacted, knowing that structurally diverse microtubules may carry different net charges.

In the present work, we demonstrate a new method to confirm the charges of molecular motors in vitro. Through implementing this method, we have successfully identified and confirmed the electric charge of one of the molecular motors, kinesin-1.

## 2. Results

In this study, we confirmed the positive electric charge of one sub-group of kinesin motors, kinesin-1, in the absence of other cellular components in vitro, by utilizing an experimental nanotechnology-based approach. In this experimental design, we developed a method to monitor the behavior of kinesin-coated microspheres under the influence of a uniform electric field.

It was particularly important to first monitor the behavior of plain microspheres inside the electric field. To conduct these control experiments, 1 to 1.2 µL of prepared beads, described in the Materials and Methods section, was transferred and confined in the space between the electrodes of the microscope slide-based nano-capacitors. The area between electrodes and consequently the beads in this area were visualized under darkfield microscopy. Upon the implementation of the DC voltage and establishing a uniform electric field between electrodes, suspended microspheres in the surrounding liquid media started to move toward the positive electrode of the capacitor. This was an indication that the beads were originally negatively charged and started to move due to the electrophoretic force.

The behavior of these plain beads in a kinesin free environment was assessed under two different implemented voltages of 1 and 1.5 volts. The uniform electric fields of 66 V/cm and 100 V/cm that can be produced between electrode gaps in the absence of any dielectric was affected, due to the dielectric specifications of the beads and the surrounding liquid media.

**Figure 1:**
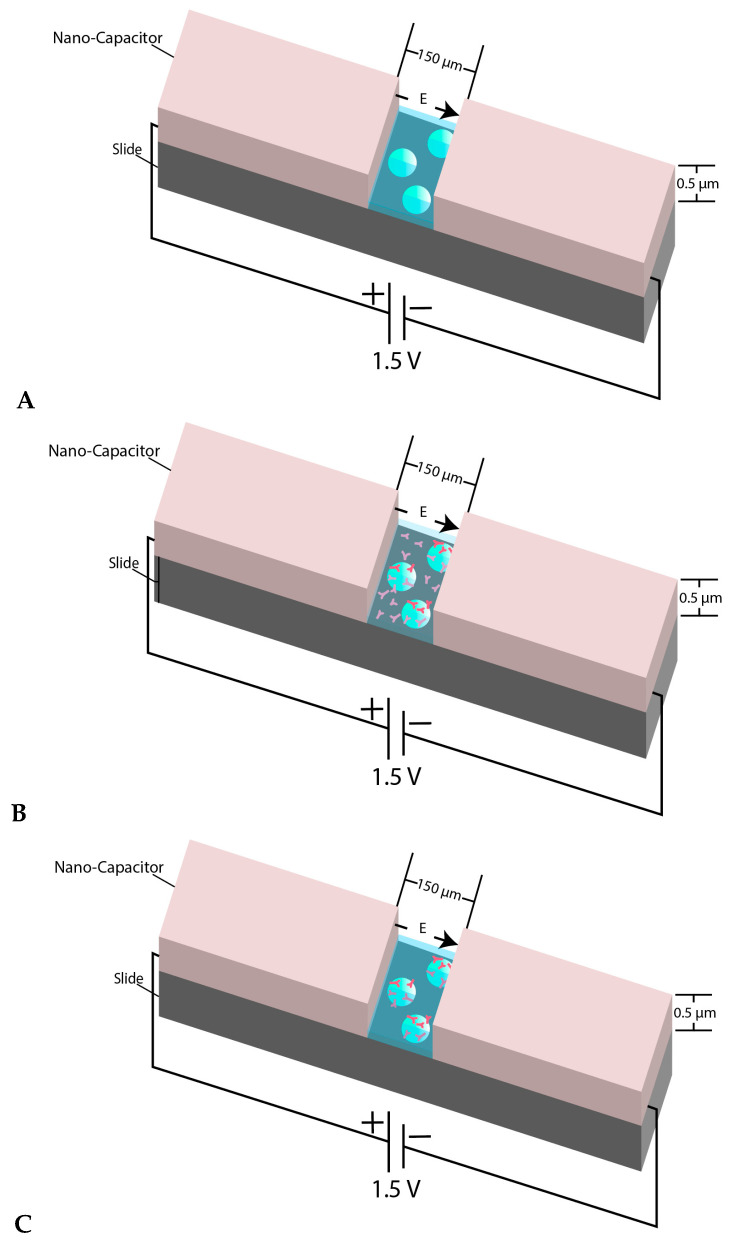
(**A**) View of the micron-sized capacitor built on a microscope slide. The space between two electrodes is 150 µm and the coating thickness is almost 0.5–0.55 µm. The behavior of microsphere plain beads was monitored inside the nanocapacitor’s gap in a kinesin free environment; (**B**) When the beads were mixed with kinesin and were immediately transferred between the nano-capacitor’s electrodes, we refer to the beads in such samples as “un-incubated beads”; (**C**) shows the schematic view of the experiment when the beads were mixed and incubated with kinesin before transferring into the capacitor’s gap. The beads in such samples are referred to as “incubated beads”. The figure’s components are not to scale.

In our experimental design, we did not quantify the dielectric constant and the actual value of the produced electric field was not precisely achievable due to the random number of beads, as well as the random density of molecular motors in the capacitor gap. While the value of the dielectric constant was not a factor in our experiment, we know that the actual value of the strength of the electric field reduces due to the presence of the media between electrodes. Figure 1 illustrates the schematic view of the three-step designed experiment.

**Figure 2:**
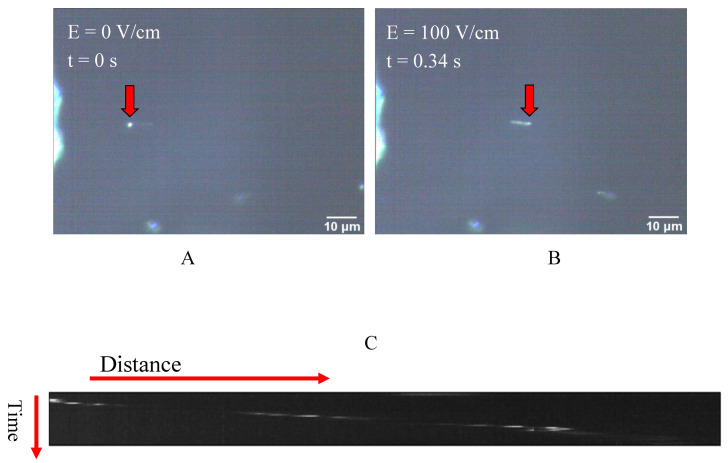
(**A**,**B**) Two represented snapshots show the movement of a bead as the electric field is implemented; (**C**) An example of a kymograph obtained from the darkfield microscopy images, which indicates the movement of the microspheres inside the capacitor and under the influence of the electric field.

In Figure 2 the movement of the beads under the influence of the uniform electric field is shown. The presented snapshots show the movement of a sample bead in the absence of kinesin protein inside the electric field, as shown in Figure 2A. The movement of the beads upon the implementation of the DC voltage and, consequently, the electric field was also confirmed by the generated kymographs obtained from different movies recorded by the microscope camera. A sample of the generated kymographs is presented in Figure 2C. It should be noted that no movement was observed in voltages below 1.0 volt.

The observed displacement of each individual bead primarily resulted from interactions of three different forces: the electrophoretic force created by the electric field on the originally negatively charged beads; the drag force on the beads due to passing through the surrounding solution; a possible dielectrophoresis force associated with the differential polarization of the object and the environment around it [20,23,24].

**Figure 3:**
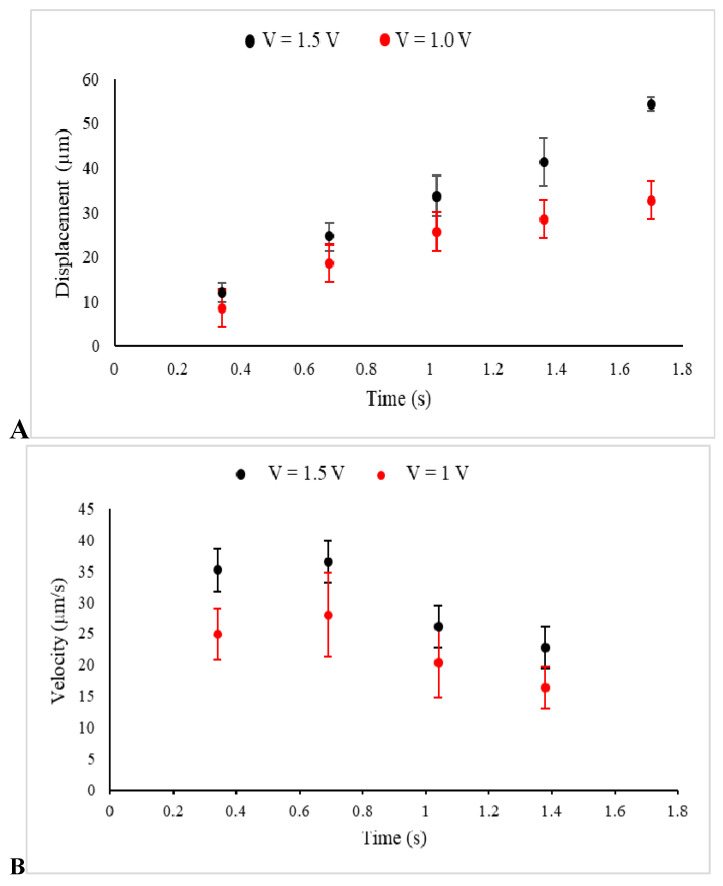
The displacements (**A**) and the velocities (**B**) of beads in a kinesin-free environment and under electric fields between electrodes, created by the voltages 1.5 V (Black) and 1 V (Red) in these sets of control experiments, are presented.

The displacement of several prepared beads, as described in Materials and Methods, under the two different implemented voltages and, consequently, two distinct uniform electric fields, was observed, recorded, and analyzed by Image J. The data collected from several individual beads were then collectively used to express displacement in terms of mean ± SEM at several sequential time frames, as shown in Figure 3A. Additionally, Figure 3B illustrates the average velocity of several beads obtained from the slope of the displacement time graph between two points.

Illustrated in Figure 3A, the beads started to move under the electrophoretic (Coulomb) force and were consequently affected by the drag force of the media, which slowed down their drifted motion, causing them to become completely immobilized or just show minor Brownian motion. Furthermore, under a stronger implemented electric field (1.5 V), the average displacement of the beads was extended. The obtained values for the average displacements at *t* = 1 s were 33.67 ± 4.6 µm (*n* = 9 measurements, 1.5 V) and 25.7 ± 4.9 µm (*n* = 8 measurements, 1V), with the two tailed *p*-value equal to 0.25. Additionally, a higher value of the velocity, which was observed in the stronger electric field was an assurance for the applicability and reliability of the designed experiment. Furthermore, the motion started with high velocity, which was reduced significantly under the effect of the drag force [25].

To explore the existent kinesin-1′s electric charge, the behavior of kinesin-coated beads under the influence of the uniform electric field was evaluated in different sets of experiments and under the same experimental conditions.

We first mixed the prepared beads with a concentration of 0.25 mg/mL kinesin-1. This mixture was then immediately used in making samples by transferring 1.2 µL of the mixture to the micro-capacitor’s gap. We refer to the beads in such samples as “un-incubated beads”. In the next set of experiments, the mixture of the kinesin-1 and the beads was incubated at room temperature for 30 min before the samples were prepared and the displacements of the beads were analyzed. Finally, we conducted the same experiment when prepared beads were incubated under the same conditions in a concentration of 0.5 mg/mL kinesin-1 protein.

The drifted motion of the beads in all experiments showed the same pattern as those obtained from the beads tested in control experiments. As the data indicate, in samples of un-incubated beads, the average displacement of those beads was reduced, compared with that calculated from the measurements of the beads in the control experiments (in the kinesin-free environment). This reduction is more likely linked to the change in several factors—the presence of kinesin-1 protein in the solution elevated the viscosity of the solution and, consequently, increased the strength of the drag force acting on the beads. In addition, the dielectric constant of the liquid environment between the two electrodes of the nanocapacitor was affected, which may cause a reduction in the strength of the electric field between two electrodes.

The average displacement for un-incubated beads after 1.0 s and under electric field produce by the voltage of 1.5 V was obtained to be 8.84 ± 1.8 µm (*n* = 5 measurements), which was significantly different from the displacement obtained from the beads in the control experiments (33.67 ± 4.6 µm) which was conducted in the absence of any kinesin-1 proteins (*p* = 0.002). The reduction in the average displacement of the un-incubated beads inside the surrounding media containing kinesin proteins is an indication that the factors explained above (viscosity and the actual electric field) were affected. We conducted the next set of experiments to confirm the charge of kinesin. To do so, the behavior of microsphere beads incubated with kinesin proteins was evaluated under the same implemented voltage of 1.5 V.

The average displacement for beads incubated in 0.25 mg/mL kinesin-1 for 30 min was obtained to be 5.7 ± 0.7 µm (*n* = 8 measurements, *t* = 1 s). As compared with the displacement of un-incubated beads after the same time, the further reduction observed in the displacement of beads in these samples was noticeable (*p* = 0.08).

In the third set of experiments, the unincubated and incubated beads in kinesin-1 protein with the concentration of 0.5 mg/mL were observed and the results of their displacements were compared with one another and with the results that were obtained from the beads incubated with the concentration of 0.25 mg/mL of the protein.

The average displacement of un-incubated beads in the samples with the higher concentration of protein was 4.52 ± 0.43 µm (*n* = 7 measurements, *t* = 1 s). This value for the incubated beads in the same protein concentration was obtained to be 3.2 ± 0.5 µm (*n* = 6 measurements, *t* = 1 s). Once again, incubated beads showed a more significant reduction in their displacements (*p* = 0.09).

Additionally, beads in higher protein concentration showed an average displacement almost equal to 40% of that observed in samples with lower protein concentration. This difference is statistically significant (*p* = 0.01 for the average displacement obtained from incubated samples in two concentrations and *p* = 0.02 for the similar parameter obtained from unincubated samples).

The beads, which were incubated with kinesin-1 for 30 min, were more inclined to be coated by the molecular motors in the solution. The comparison between the displacement of the un-incubated beads and incubated beads can now be associated with the molecular motors attached to the beads.

**Figure 4:**
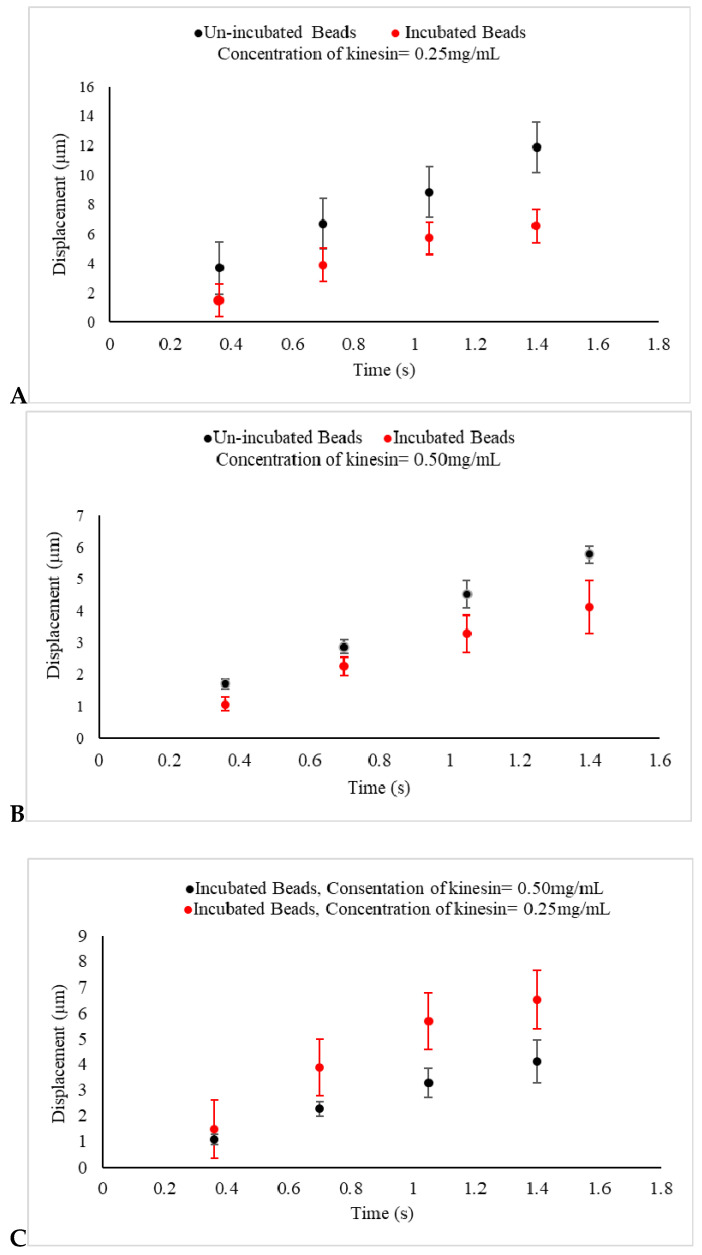
(**A**,**B**) The displacements of un-incubated kinesin beads (Black) and incubated beads in 0.25 mg/mL and 0.5 mg/mL kinesin-1 (Red) under an electric field produced by 1.5 V are presented. In un-incubated samples in both concentrations, the displacements of beads are reduced, as compared with beads in the control experiments, as shown in Figure 3A. The displacements of incubated beads are also seen to be reduced, as compared with un-incubated beads under the same experimental conditions. This indicates that the negative charge of prepared beads used in the control experiment is decreased because of the positive charge of kinesin proteins adhering to the beads. As expected, the reduction in displacement is more significant as the concentration of the protein increases; (**C**) The displacement of kinesin-coated beads, incubated in 0.25 mg/mL (Red) and 0.5 mg/mL kinesin protein (Black) for 30 min and under the same uniform electric field, are presented. This indicates that the negative charge of prepared beads is decreased even more by the greater concentration of the positive charge of kinesin proteins adhered to the beads.

A shorter drifted displacement of the kinesin incubated beads, as shown in Figure 4A,B, as compared with the un-incubated beads is evidence that the electrophoretic force acting on the beads produced by the electric field was weaker. This change on the force can now be linked to the decrease in the net distribution of the original negative surface charge of the beads. This reduction can be caused by the positive charge that motor proteins attached to the beads carry. This is an indication that motors carry positive electrostatic charge, a confirmation through a nanotechnology-based approach in vitro, which is in consistent with other studies [26].

The addition of the kinesin-1 proteins had a major effect in this series of experiments. First, the obtained evidence suggests that the shorter drifted displacement, caused by the regulated net force acting on the beads, was tied to the charge that the kinesin-1 carried. Second, the observed results from the last set of experiments, as shown in Figure 4C, when the beads were incubated in the higher concentration of kinesin-1, represented an even shorter displacement in samples under the influence of an electric field. Collectively, this evidence confirms that kinesin-1 proteins carry positive electric charge, a validation through an in vitro method, which can be utilized to evaluate the electric charge of other molecular motors as well.

## 3. Discussion

The interplay between molecular motors and microtubules is identified as a contributory factor that allows molecular motors to bind to microtubules and use them as tracks for intracellular functions, including cellular transportations. The electrostatic interaction is known as a factor that enables the kinesin–tubulin association. As microtubules carry negative net charge, the charge of molecular motors is concluded to be positive to make the binding of molecular motors to microtubules possible. However, the knowledge about the electrostatic charge of molecular motors in vitro and in the absence of microtubules and other cellular proteins has been poorly investigated. We initiated this experimental design to confirm the charge of biomolecular motors in vitro, where the effects of other cellular components can be eliminated. This new approach was based on monitoring the behavior of kinesin-coated microsphere beads under the influence of a uniform electric field. The observational results confirm that this approach can be considered a method to detect the charge of molecular motors. The method presented in this work can be easily expanded to optimize the experimental conditions for broader applications in detecting and confirming the possible charge of other motors or cellular components.

This experimental design, which relies on our capabilities in the area of nanotechnology, provides us with a better insight about the biophysical properties of biomolecular motors. This method paves the way for further in-depth studies of the functions and properties of bio-molecular motors.

## 4. Materials and Methods

In a set of parallel studies, we observed and analyzed the behavior of kinesin-1-coated microspheres in a uniform electric field in vitro. Each component of our experimental setting is described below.

### 4.1. Microscope Slide-Based Micron-Sized Capacitor

To create a uniform electric field, a microscope slide-based capacitor was used. In this design, a microscope slide was coated by Indium–Tin Oxide (ITO). The thickness of the ITO was almost 5500 Å. The uncoated gap created in the center of the microscope slide was 150 µm (Deposition Research Lab, Inc., St. Charles, MO, USA). Upon connection to a DC power supply, a uniform electric field was created in the gap of the micron-sized capacitor built on each microscope slide.

The implemented voltage in this study was 1.5 volts (under our experimental conditions, the higher implemented voltages were damaging the capacitor). However, in our controlled experiments the implemented voltages were 1 and 1.5 volts, as explained in the next section. The magnitudes of the electric fields in the absence of a dielectric material under these voltages were almost 66 V/cm and 100 V/cm accordingly.

### 4.2. Silica Microsphere Beads

The Silica microspheres employed in this experiment was purchased from Bangs Laboratories, Inc. (Fishers, IN, USA). The Sio2 microsphere (beads) were available as an aqueous suspension. The diameter of individual beads was 0.5 µm with a density of 2 g/cm^3^. Due to the high concentration of beads, they were diluted 10^4^ times by serial suspension in purified water. We conducted parallel experiments under the same experimental conditions. Therefore, no attempt was made to wash the beads and separate them from their liquid phase prior to the serial dilution of the beads. We refer to these beads diluted by water as “prepared beads”.

### 4.3. Protein Purification and Sample Preparation

Kinesin-1 was obtained from Cytoskeleton Inc. (Denver, CO, USA). The lyophilized kinesin-1 (Kinesin Heavy Chain Motor Domain, Cytoskeleton, Co., Cat. #KR01) was first re-suspended in purified water to achieve the concentration of 1 mg/mL, divided to small allocations, and stored at −20 °C. To reach a lower concentration of kinesin at the time of the experiment, each allocation was mixed with prepared beads (explained in the previous section). The finalized concentration of molecular motors used was 0.25 mg/mL and 0.5 mg/mL. The mixture of molecular motors–beads was either immediately used or was incubated for 30 min at room temperature prior to being used. This incubation time created an environment for the molecular motors to attach to the beads. The 1–1.2 µL of these un-incubated kinesin-beads or incubated kinesin beads were then transferred to the micro-capacitor. The sample was then covered by a clean coverslip (Ted Pella, Redding, CA, USA, thickness No. 0) and completely sealed with vacuum grease, which created a confined geometry with inner glass, surfaced around 5 µm. Through this procedure, beads were randomly distributed in the created enclosed area. Among them, some of the beads fell in the 150 µm area between two electrodes of the microscope slide-based capacitor. The beads that fell between two electrodes of the capacitor were visualized and the behavior of the beads in that area was monitored, as explained below.

It should be emphasized that the goal of the experiment was to confirm the nature of the charge of kinesin proteins. To achieve this goal, we eliminated the interaction of kinesin proteins with other potentially charged proteins by preparing samples in the absence of any blocking solutions (such as casein solution). This choice of experiment had two effects: it increased (a) the possibility of un-wanted binding of protein coated beads with each other and consequently the formation of the clumping of the beads; (b) the immobilization of the beads due to un wanted adhesion to the surface of the microscope slide.

The majority of the beads in the samples were clumped after 40–50 min of the incubation. This was an indication that the beads were covered by kinesin protein. To observe the behavior of single beads, with a reasonable level of certainty that the beads were incubated long enough to be coated by the protein, the mixture of protein–beads was ideally incubated for 30 min.

In addition, the nano-capacitors could easily burnout by increasing the voltage. As we added the kinesin protein, no movement was observed in the initial voltage of 1 volt at the presence of 0.25 mg/mL or less of kinesin proteins. Our experimental conditions were adjusted by increasing the voltage to 1.5 volts. At this set voltage, we could repeatedly observe the change, which resulted in the displacement of the beads in the presence of kinesin in different samples. However, while we could potentially see the bead movements in the presence of lower protein concentration with increasing the voltage, this approach was not applicable, due to the limitation of the functionality of the capacitors under higher voltages. We did not observe the beads’ movements in the concentrations higher than 5 mg/mL, mainly due to the binding of the beads to the glass surface or one another. The choice of protein concentrations in this study provided us with the experimental frame that the beads’ movements and any changes in their movements were significant and were observed under our experimental limitations.

Further, the average diameter of the beads after the incubation with the high concentration of kinesin protein was measured (implementing imageJ, U.S. National Institute of Health, Bethesda, MD, USA, https://imagej.nih.gov/ij/) to assess any possible changes in the size of the beads due to the coverage with the protein. In incubated samples with 0.5 mg/mL of protein, the average diameter obtained was 0.52 ± 0.006 µm, which is comparable with the original sizes of the prepared beads. Therefore, no evidence of significant changes in the sizes of the beads after the incubation was observed.

### 4.4. Visualization and Analysis

In this experiment we used a Nikon upright microscope (Nikon Instruments Inc., Melville, NY, USA), equipped with a 100×/1.25 NA oil immersion objective lens, and a 1.43–1.20 oil dark-field condenser. The microscope was connected to a Lumenera- Infinity 1-3C camera with Infinity Analyze software (Teledyne Lumenera, Ottawa, ON, Canada). The movement of the individual beads was then recorded through video microscopy. The movies taken through this procedure were then analyzed by ImageJ (Rasband, W. S, ImageJ, National Institutes of Health, Bethesda, MD, USA).

In our study, we measured the displacement of the different beads. However, as explained above, we experienced the unwanted binding of the beads with the microscope slide surface in the absence of the blocking solutions. Therefore, to obtain data, we relied on one-time recording of the movement of a single bead under the uniform electric field. Therefore, our data are obtained from several samples prepared under the same experimental conditions. After collecting the data from different samples, the mean ± STEM of the displacements was calculated. Consequently, the associated *p* value was then obtained.
